# Improved inflammatory bowel disease, wound healing and normal oxidative burst under treatment with empagliflozin in glycogen storage disease type Ib

**DOI:** 10.1186/s13023-020-01503-8

**Published:** 2020-08-24

**Authors:** Sarah C. Grünert, Roland Elling, Bärbel Maag, Saskia B. Wortmann, Terry G. J. Derks, Luciana Hannibal, Anke Schumann, Stefanie Rosenbaum-Fabian, Ute Spiekerkoetter

**Affiliations:** 1 Department of General Paediatrics, Adolescent Medicine and Neonatology, Medical Centre- University of Freiburg, Faculty of Medicine, Mathildenstraße 1, 79106 Freiburg, Germany; 2 University Children’s Hospital, Paracelsus Medical University (PMU), Salzburg, Austria; 3 Radboud Center for Mitochondrial Medicine, Department of Pediatrics, Amalia Children’s Hospital, Radboudumc, Nijmegen, The Netherlands; 4 Section of Metabolic Diseases, Beatrix Children’s Hospital, University Medical Centre Groningen, University of Groningen, Groningen, The Netherlands; 5 Department of General Paediatrics, Adolescent Medicine and Neonatology, Laboratory of Clinical Biochemistry and Metabolism, Medical Centre-University of Freiburg, Faculty of Medicine, Freiburg, Germany

**Keywords:** Glycogen storage disease type Ib, Neutropenia, Neutrophil dysfunction, Empagliflozin, Wound healing, Inflammatory bowel disease, Oxidative burst, Glucose-6-phosphate transporter

## Abstract

**Background:**

Glycogen storage disease type Ib (GSD Ib) is a rare inborn error of glycogen metabolism due to mutations in *SLC37A4*. Besides a severe form of fasting intolerance, the disorder is usually associated with neutropenia and neutrophil dysfunction causing serious infections, inflammatory bowel disease, oral, urogenital and perianal lesions as well as impaired wound healing. Recently, SGLT2 inhibitors such as empagliflozin that reduce the plasma levels of 1,5-anhydroglucitol have been described as a new treatment option for the neutropenia and neutrophil dysfunction in patients with GSD Ib.

**Results:**

We report on a 35-year-old female patient with GSD Ib who had been treated with G-CSF for neutropenia since the age of 9. She had a large chronic abdominal wound as a consequence of recurrent operations due to complications of her inflammatory bowel disease. Treatment with 20 mg empagliflozin per day resulted in normalisation of the neutrophil count and neutrophil function even after termination of G-CSF. The chronic abdominal wound that had been unchanged for 2 years before the start of empagliflozin nearly closed within 12 weeks. No side effects of empagliflozin were observed.

**Conclusion:**

SGLT2 inhibitors are a new and probably safe treatment option for GSD Ib-associated neutropenia and neutrophil dysfunction. We hypothesize that restoration of neutrophil function and normalisation of neutrophil apoptosis leads to improvement of wound healing and ameliorates symptoms of inflammatory bowel disease.

## Background

Glycogen storage disease type Ib (GSD Ib) is a rare disorder of glycogen metabolism due to mutations in *SLC37A4* encoding the glucose-6-phosphate transporter of the endoplasmic reticulum. This glucose-6-phosphate transporter is ubiquitously expressed and transports glucose-6-phosphate from the cytosol to the lumen of the endoplasmic reticulum where it can be hydrolyzed by glucose-6-phosphatase [1]. The estimated prevalence of GSD Ib is about 1: 500,000 [2]. GSD Ib is clinically characterized by severe fasting hypoglycemia, hepatomegaly, failure to thrive, growth retardation, truncal obesity, doll-like facies, short stature, bleeding tendency and hypotrophic muscles [3]. Laboratory findings include hyperuricemia, hyperlipidemia, and elevated lactate levels [3]. Additionally, most patients with GSD Ib develop neutropenia and inflammatory bowel disease. Neutropenia is the hallmark feature of GSD Ib, however, the age at onset as well as the clinical course are variable [3]. It may be present already at birth or not appear until late in childhood as cyclic or permanent neutropenia [3, 4].

Polymorphonuclear neutrophils (PMNs) from patients with GSD Ib are not only reduced in number, but also dysfunctional [4]. Neutropenia is caused by both a decrease in the production of mature neutrophils as well as enhanced neutrophil apoptosis [5–7]. PMN dysfunction is reflected by impaired respiratory burst, chemotaxis, and calcium mobilization activities [8, 9]. G6PT-deficient PMN show reduced glucose utilization characterized by decreased glucose uptake and reduced levels of intracellular G6P, lactate, adenosine triphosphate, and reduced NAD phosphate [5]. This energy impairment is likely responsible for their decreased capacity to produce superoxide, decreased protein glycosylation, and increased endoplasmic reticulum stress [10]. Deficiency of G6PT also causes impairment in neutrophil adhesion and migration via aberrant expression of β2 integrins [11].

**Fig. 1:**
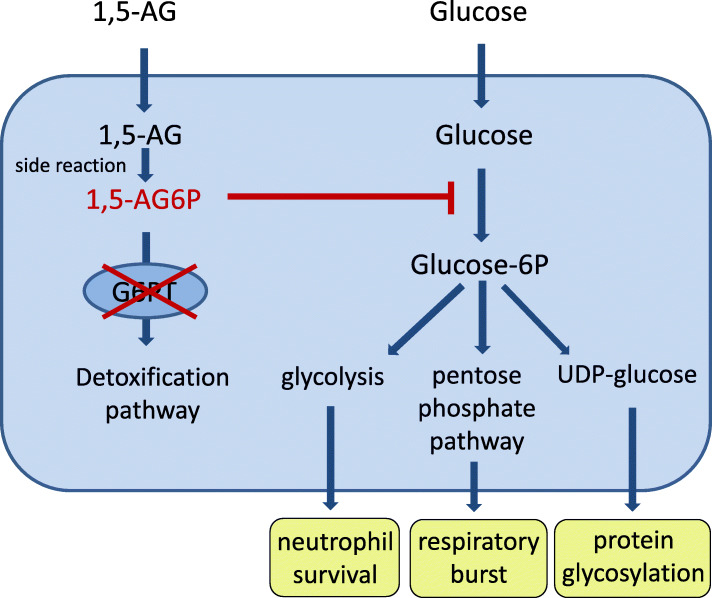
Mechanism of neutropenia in glycogen storage disease type 1b. 1,5-anhydroglutitol, a non-degradable glucose analogue, is phosphorylated to 1,5-anhydroglucitol-phosphate. This metabolite is usually detoxified by transport to the endoplasmic reticulum by the glucose-6-phosphate transporter (G6PT) and subsequent dephosphorylation by the enzyme G6PC3. In patients with GSD Ib who are deficient in G6PT, 1,5-anhydroglucitol-phosphate accumulates in toxic concentrations. This metabolite is a strong inhibitor of hexokinases resulting in depletion of the intracellular glucose-6-phophate pool that is vital for normal survival and function of the neutrophils

Veiga-da-Cunha et al. have recently shown that the impaired glucose utilisation of G6PT-deficient neutrophils is caused by failure to eliminate 1,5-anhydroglucitol-6-phosphate (1,5-AG6P), a close structural analogue of glucose-6-phosphate [10] (Fig. 1). Accumulation of 1,5-AG6P within the granulocytes strongly inhibits the activities of hexokinases that catalyse the first step of glycolysis, resulting in energy deficiency of the cells and apoptosis (Fig. 1). Studies in a G6PC3-deficient mouse model that phenotypically and biochemically mimics the PMN impairment of patients with GSD Ib have shown that treatment with an inhibitor of the kidney sodium glucose co-transporter 2 (SGLT2) was able to lower the blood level of 1,5AG6P and consequently restore a normal neutrophil count [10]. This has already prompted first treatment trials in single GSD Ib patients with positive effects, such as improvement of inflammatory bowel disease, ulcerative stomatitis and anemia [12]. SGLT2 inhibitors such as empagliflozin are anti-diabetic drugs that inhibit renal glucose reabsorption resulting in an increased urinary excretion of glucose [13]. Glucosuria decreases renal 1,5-AG reabsorption and thereby lowers its serum concentrations [12, 14]. Treating patients with a disorder associated with fasting hypoglycemia with a glucose-lowering drug might be counterintuitive, however, no severe side effects have been reported in the first few treated patients so far [12]. We report on a 35-year-old female patient with neutropenia, inflammatory bowel disease, massive splenomegaly and thrombocytopenia after 26 years of granulocyte-colony stimulating factor (G-CSF) treatment and a severe wound healing defect after abdominal surgery who showed remarkable improvement of wound healing and neutrophil count and function upon treatment with empagliflozin.

## Methods

### PMN isolation and apoptosis assays

Neutrophil granulocytes were isolated from peripheral blood using density-gradient centrifugation. Equal number of controls or patient cells were cultured in RPMI supplemented with 10% FCS plus 0.5% Ciprofloxacin and in some experiments exposed to GM-CSF (100 ng/ml; PeproTech) or Q-VD  (1 μM; MP Biomedicals). After 0, 6 and 12 h apoptosis was analyzed by Annexin V/ Propidium iodide staining (BD Pharmingen / BioLegend) on a Gallios flow cytometer. Purity of neutrophils was measured by staining for CD66b (BD Pharmingen) and typically yielded > 99% positive cells.

### Oxidative burst

Reactive oxygen species of PMN were measured using the DHR assay as described by Vowells et al. [15]. Briefly, leucocytes were loaded with dihydrorhodamine 123 (DHR-123, Sigma) after red blood cell lysis at 37 °C for 5 min and stimulated with phorbol myristate acetate (PMA) for 15 min. The samples were immediately analyzed by flow cytometry using the FL-2 channel (585 nm).

## Results

### Case report

The patient is a 35 year-old woman who was diagnosed with GSD Ib at the age of 10 months. Dietary treatment was started with good metabolic control. Since the age of 9 years the patient has received G-CSF treatment for neutropenia and recurrent infections. Since the age of 10 she has suffered from inflammatory bowel disease. The clinical course was further complicated by recurrent abdominal abscesses. At age 25, subtotal colectomy was performed due to long-distance stenosis of the transverse colon with an abscess in the right upper abdomen. Postoperative wound healing was impaired and required surgical wound revision and closure. Pancreatic fibrosis was observed at age 28, exocrine pancreatic insufficiency was diagnosed at age 31, and supplementation with pancreatin was initiated. Hepatosplenomegaly was observed, and the thrombocyte count was significantly reduced to around 40 G/l, possibly due to the massive splenomegaly. At age 33, the patient presented with acute abdominal pain in the lower abdomen. MRI was suggestive of a pelvic abscess, enlarged pelvic retroperitoneal and mesenterial lymph nodes as well as inflammatory alterations of the intestine. The patient had bloody stools, and her overall clinical condition was reduced. Antibiotic therapy with meropenem, ampicillin and metronidazole was started. Explorative laparotomy with extensive resection of the small bowel (side-to-side jejuno-jejunostomy 70 cm distal of the ligament of Treitz and end-to-end jejuno-jejunostomy 150 cm distal of the ligament of Treitz, resection of the stenosing ileodescendostomy) was performed. Abdominal bleeding occurred in the postoperative course, which required 4 further laparotomies within 3 weeks. Secondary surgical closure of the wound could be performed 4 weeks after the initial operation. However, within the consecutive weeks a large wound dehiscence developed with severely impaired wound healing. At its maximum the wound had a diameter of 29 × 18 cm. Surgical revision of the abdominal wall defect after 1 year was not of permanent success. A colonoscopy was performed alongside this procedure, which revealed mild to moderate inflammatory activity. Supplementation with protein powder as well as arginine and glutamine was started to promote wound healing, but was also without success.

**Fig. 2:**
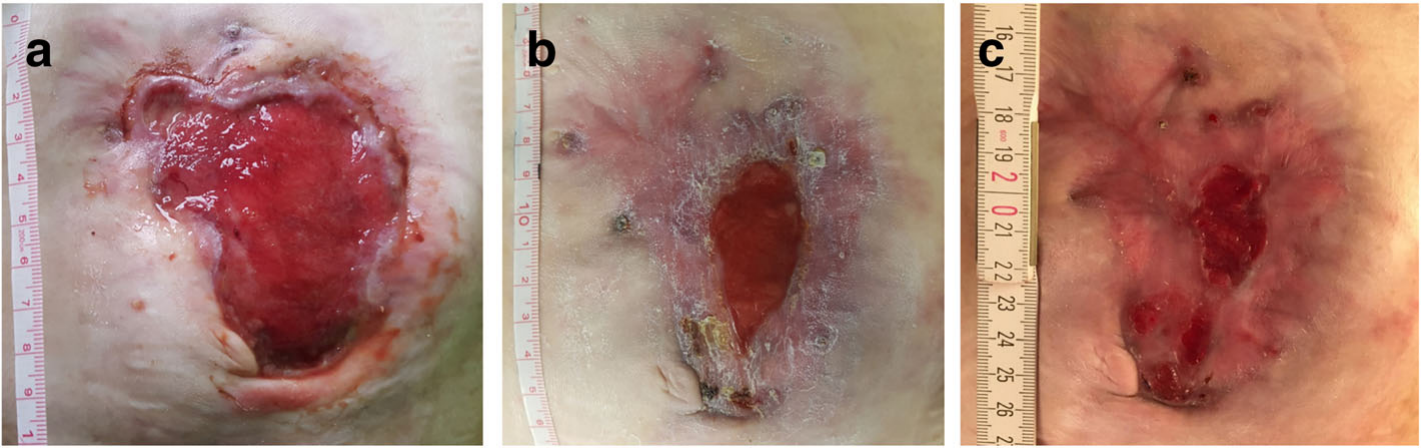
Development of the abdominal wound under empagliflozin treatment. **a** Before start of empagliflozin treatment **b** on day 50 on empagliflozin and **c** on day 85 on empagliflozin

At age 35 the patient first presented to our metabolic clinic. Her body weight was 50 kg, body length 153 cm and BMI 21.4 kg/m^2^. She was in good metabolic control under a GSD diet with regular meals every 2 h during the day and continuous nocturnal feeding with tube feeding formula and maltodextrin (composition of diet assessed by a 3-day dietary protocol: carbohydrates 47% of energy intake, fat 34% of energy intake, and protein 18% of energy intake). Continuous glucose monitoring was performed using a Dexcom G6 device. Transaminase activities (AST 13 U/L, ALT 19 U/L, normal 10–35 U/L), serum triglycerides 140 mg/dL (< 150 mg/dL), uric acid 5.4 mg/dl (2,4–5,7 mg/dL) and lactate 1.0 mmol/L (normal 0,5–1,6 mmol/L) were normal. Total protein, albumin and pre-albumin concentrations in serum were also within the respective reference ranges (7.6 g/L (normal 6.4–8.3 g/L), 4.2 g/L (normal 3.5–5.2 g/L) and 0.21 g/L (normal 0.2–0.4 g/L), respectively). Abdominal ultrasound still showed massive hepatosplenomegaly (liver 17.8 cm, normal < 15 cm; spleen 27.1 cm, normal < 14 cm). Thrombocyte count was severely reduced with 42 G/L, whereas the hemoglobin level was normal at 11.6 g/dL. The patient had about 10–11 loose to watery stools per day. The Crohn’s Disease Activity Index (CDAI) was 398. This index is the gold standard to determine current activity of Crohn’s disease. A CDAI score < 150 is defined as remission of Crohn’s disease, while a value of greater than 450 is defined as severe disease. The Crohn-like disease was treated with budesonide 3 mg/day, 5-aminosalicylic acid 3 × 1 g/day, vitamin E 800 mg/day and loperamide 2 mg. Stool calprotectin was within the normal range (42 mg/kg, normal < 50 mg/kg), CRP was normal (0.7 mg/dl, normal < 1 mg/dl), and the blood sedimentation rate was 25 within the 1st hour (normal < 20 mm). The clinical condition was stable but the overall well-being was markedly impaired, the appetite was reduced. The abdominal wall defect was 8 cm × 6.5 cm and oozing (Fig. 2). Colonoscopy or capsule endoscopy was not performed due to the high risk of abdominal complications. Anogenital lesions had not occurred within the last 20 years, oral lesions only after special triggers such as dental cleaning.

**Fig. 3:**
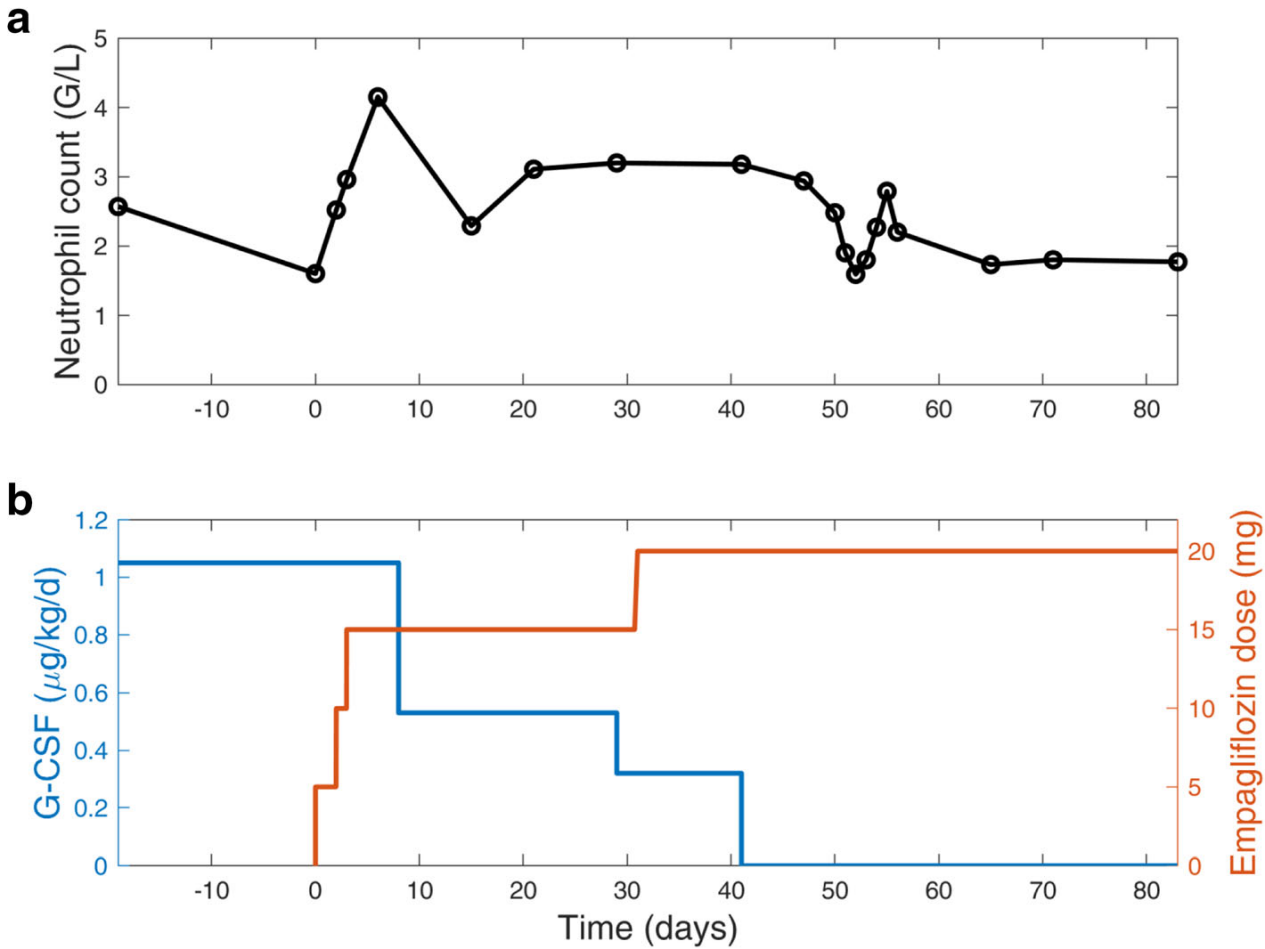
Absolute neutrophil count, G-CSF dose and empagliflozin dose over the time of treatment. **a** Absolute neutrophil count. **b**) G-CSF and empagliflozin dose. The G-CSF dose was reduced stepwise and G-CSF could be discontinued after 26 years of treatment on day 41 on empagliflozin

After informed consent for this individual treatment the patient was admitted for the initiation of empagliflozin therapy. The starting dose was 5 mg/day, and the dose was subsequently increased to 2 × 5 mg/day on day 2, and 10 mg/5 mg on day 3. Absolute neutrophil count at admission was 1600/μl under 1.05 μg G-CSF/kg/day. The dietary regimen was continued as usual, and no relevant hypoglycemias were observed. The minimal blood sugar was 4.3 mmol/L. The patient could be discharged on day 4. Within the following weeks the neutrophil count increased, and the G-CSF dose could subsequently be decreased stepwise as shown in Fig. 3. The appetite and well-being improved. The stool frequency also decreased to 5 to 7 stools per day, and loperamide could be terminated. Wound-healing distinctly improved and the abdominal wall defect slowly granulated (Fig. 2). After 1 month, the empagliflozin dose was increased to 2 × 10 mg/day. Administration of GCSF was terminated 41 days after the start of empagliflozin (Fig. 3). No major side effects of empagliflozin were observed, such as urogenital infections, hypoglycemia and ketoacidosis. Albumin concentration remained stable within the normal range (4.2 g/L, normal 3.5–5.2 g/L). The body weight remained stable during the treatment period, and no relevant change in thrombocyte count or haemoglobin level was observed. The CDAI score decreased to 184 on day 50 of empagliflozin treatment. Fasting tolerance also slightly increased. The wound size on day 85 of treatment was about 3 × 1.5 cm.

**Fig. 4:**
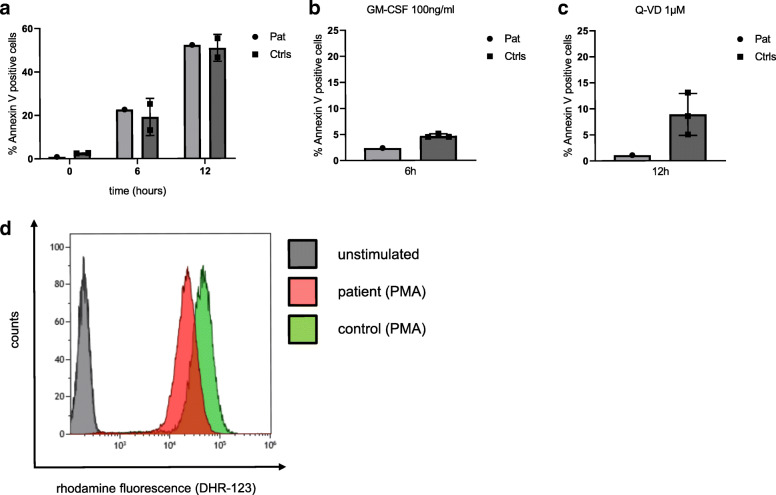
Apoptosis and ROS production of patient and control PMN. **a** Percentage of apoptotic PMN (Annexin V positive) after 0, 6 and 12 h culture showing equal rates of apoptotic cells for healthy donors and patient. **b** percentage of apoptotic PMN (Annexin V positive) in healthy donors and patient after 6 h culture in the presence of 100 ng/ml GM-CSF. **c** Fraction of apoptotic cells (Annexin V positive) collected from healthy donors and patient after 12 h of caspase inhibition with 1 μM Q-VD. **d** ROS production in patient and healthy donor PMN measured by rhodamine fluorescence. Fluorescence intensity of DHR123-loaded PMN in basal conditions (PBS) or after activation by PMA

As PMN from GSD Ib patients are known to show impaired ROS production and an increased apoptosis phenotype, we analysed both apoptosis as well as reactive oxygen production in neutrophils from our patient (Fig. 4) on day 50 of empagliflozin treatment. As shown in Fig. 4a, spontaneous apoptosis of isolated PMN did not differ compared with healthy control cells. Furthermore, apoptosis could be efficiently reduced by supplementation of cell culture media with GM-CSF (Fig. 4b). PMN apoptosis could be fully abrogated by the pan-caspase inhibitor Q-VD, an effect which was even more pronounced in patient PMN compared to control cells (Fig. 4c). We next measured reactive oxygen species of patient and control PMN after PMA stimulation and observed no significant differences, further confirming the stabilized phenotype of patient PMN under treatment with empagliflozin.

## Discussion

For more than 25 years, the standard therapy for neutropenia associated with recurrent infections in GSD Ib patients has been the supplementation of G-CSF. G-CSF is effective to raise blood neutrophil counts and to reduce infections in most patients [16]. However, doses must be limited due to side effects such as increases of spleen size with abdominal pain and hypersplenism. Long-term G-CSF treatment may also result in an increased risk of myelodysplastic syndrome (MDS) or acute myeloid leukemia (AML) possibly due to increased marrow stress resulting in telomere shortening [2]. Several cases of AML/MDS after treatment with G-CSF for 6–25 years have been reported [2, 16–18].

After the elucidation of the mechanism of neutrophil phenotype in GSD Ib SGLT2 inhibitors emerged as a new treatment option for neutropenia and neutrophil dysfunction in GSD Ib [10, 12]. These anti-diabetic drugs that have been approved for the treatment of diabetes type 2 in adults have a favourable safety profile. Stimulation of renal glucosuria along with reduced reabsorption of 1,5-AG decreases plasma concentrations of this toxic metabolite, thus minimizing neutrophil dysfunction in GSD Ib. Wortmann et al. have reported the first four patients treated with empagliflozin and observed dramatical improvement of the heterogeneous clinical findings related to neutrophil dysfunction in all four patients [12]. This included abdominal pain, symptoms of inflammatory bowel disease, oral and anourogenital lesions and infections, dependence on tube feeding and anemia [12].

In consistence with these observations, treatment with empagliflozin resulted in a striking clinical improvement in our patient: 1) The abdominal wound that persisted for more than 2 years despite treatment with G-CSF, arginine, glutamine and protein supplementation almost closed within few weeks, 2) The frequency of stools decreased and treatment with loperamide could be stopped, the CDAI decreased to 184, and 3) the overall well-being as well as the appetite increased.

The CDAI is a score to determine the current activity of Crohn’s disease. The frequency of soft or liquid stools is one important component used in the calculation of this score. As our patient has a short bowel syndrome and is lacking large parts of her colon, she will probably not be able to produce solid stools even in the absence of inflammatory bowel disease. Therefore, the CDAI may overestimate the inflammatory activity of her Crohn’s disease.

The observed clinical improvement under empagliflozin was accompanied by a dramatic amelioration of laboratory parameters: The neutrophil count initially increased despite reduction and finally termination of G-CSF. Even without G-CSF the neutrophil count stabilized well within the reference range with an average number of neutrophils of about 2000–2500/μl. Neutropenia in GSD Ib is thought to be caused by enhanced apoptosis [6]. Apoptosis studies performed in our patient showed no evidence of increased apoptosis reflected by an equal percentage of Annexin V positive PMNs after up to 12 h culture compared with healthy donor cells. Likewise, we did not observe any evidence for impaired ROS production in patient PMNs as compared to control cells. Since neutrophils of GSD Ib patients are known to have impaired oxidative burst and bactericidal activity [5], we interpret this finding as the effect of empagliflozin therapy although we did not measure ROS production or apoptosis before initiation of this treatment.

Although the empagliflozin dose was progressively increased to 20 mg per day we observed none of the known side effects such as hypoglycemia or urinary tract infections. The daily dose of 20 mg corresponds to 0.4 mg/kg/day and it is within the dosage window used in the patients treated by Wortmann et al. between 0.3 and 0.7 mg/kg/day [12]. A clear dose-response as observed in one patient reported by Wortmann et al. could not be seen in our patient when the dose was increased from 15 mg to 20 mg/day.

Splenomegaly as seen in our patient is a common finding in GSD Ib. A large cohort study on GSD Ib including both pediatric and adult patients revealed that splenomegaly was present in 47% prior to G-CSF and 76% on G-CSF treatment [16]. Some patients showed a dramatic increase of spleen size associated with abdominal pain under G-CSF [16, 19, 20]. Several patients required splenectomy because of the degree of splenic enlargement and pain [16]. In contrast, thrombocytopenia, as observed in our patient for years, is an uncommon finding. Although it is etiologically not fully understood, it is well conceivable that it is at least partially caused by sequestration of thrombocytes by the extremely enlarged spleen. The follow-up time, however, is still too short to evaluate if splenomegaly will be reversible after the termination of G-CSF treatment and if the thrombocyte count will consecutively increase.

Empagliflozin is not approved for the treatment of neutropenia in GSD Ib and its use for this indication is off label. As treatment data are only available for very few patients so far, there is still an important gap of knowledge with respect to the risks and potential adverse effects in this population. Due to their metabolic defect, GSD Ib patients are especially prone to some of the common side effects of empagliflozin, such as hypoglycemia and urogenital infections. Additionally, chronic diarrhea associated with inflammatory bowel disease may predispose patients to dehydration. To our knowledge, no severe side effects of empagliflozin have been reported in GSD Ib so far, but systematic data collection on the safety and efficacy is necessary to further evaluate the risks and benefits of this new treatment approach.

## Conclusion

Although data on clinical and laboratory outcome parameters are still very limited, empagliflozin is a promising new option to treat neutropenia and neutrophil dysfunction in patients with GSD Ib. To facilitate systematic data collection on the safety and efficacy of this new treatment approach a patient registry has already been initiated. Further studies are needed to assess the long-term risks and benefits of this new treatment. Data from preliminary interventions indicate that empagliflozin seems to be superior to G-SCF with respect to the correction of neutrophil dysfunction and possible side effects.

## Data Availability

Not applicable.

## Abbreviations

AML: Acute myeloid leukemia
CDAI: Crohn’s Disease Activity Index
G-CSF: Granulocyte colony stimulating factor
GSD Ib: Glycogen storage disease type Ib
MDS: Myelodysplastic syndrome
PMN: Polymorphonuclear neutrophils
SGLT2: Sodium glucose co-transporter 2
